# Effects of icariin as a feed additive on the reproductive function in bucks (*Capra hircus*)

**DOI:** 10.3389/fvets.2024.1467947

**Published:** 2024-11-06

**Authors:** Fang-e Zhao, Hong Chen, Shuo Wang, Xinge Zhang, Na Chen, Hongbo Chen, Jie Fu, Hailong Liu, Jun Liu, Tengfei Liu

**Affiliations:** ^1^College of Veterinary Medicine, Northwest A&F University, Yangling, China; ^2^Institute of Animal Science and Veterinary Medicine, Hainan Academy of Agricultural Sciences, Haikou, China

**Keywords:** icariin, testes, spermatogenesis, testosterone, dairy goat

## Abstract

Improving the reproductive ability and fertility of male ruminants is a central concern in animal husbandry. Phytogenic feed additives, known for their anti-inflammatory, antioxidant, and immunomodulatory properties, are commonly used in animal feed. Icariin (ICA), the primary flavonoid glucoside derived from Epimedium, is a traditional tonic in Chinese herbal medicine. However, its potential to enhance the reproductive performance of male ruminants remains unclear. In this study, twelve healthy adult male dairy goats were divided into two groups. The goats received oral administration of ICA at doses of 0 (control) and 50 mg/kg body weight daily for a consecutive period of 80 days during the breeding season. The effects of ICA on the reproductive performance was analyzed by histological examinations, semen quality analysis, and ELISA experiments. ELISA results showed a progressive increase in serum levels of GnIH, LH, and testosterone with the prolonged ICA treatment (*p* < 0.05). However, the serum concentration of GnRH in the ICA group initially increased, followed by a subsequent decrease (*p* < 0.05). The hypothalamic concentrations of dopamine (DA) and 5-hydroxytryptamine (5-HT) were significantly higher in the ICA group compared to the control group (*p* < 0.01). The CASA system analyzed sperm kinematics and revealed that ICA increased ejaculate volume, with both total motile and progressive motile sperm gradually increasing over time (*p* < 0.05). ICA did not affect the body weight of the goats but significantly increased the organ coefficient of the testes (*p* < 0.01). Additionally, there was an upregulation of hormone receptor expression in testicular tissue and an improvement in the antioxidant capacity of the testes after ICA treatment (*p* < 0.01). Furthermore, ICA was implicated in testosterone synthesis by modulating the expression of key enzymes associated with steroidogenesis and promoting the differentiation of spermatogonial stem cell to enhance spermatogenesis. In conclusion, our results indicate that icariin, as a phytogenic feed additive incorporated into the diet of ruminants, offers potential benefits in improving the reproductive performance of male dairy goats.

## Introduction

The reproductive performance of ruminants is of great economic importance, and its improvement remains a priority in animal husbandry (1). Dairy goat (*Capra hircus*), are globally recognized as economically valuable herbivores. Buck semen plays a crucial role in successful female pregnancy and is a key factor in enabling genetic improvement within the population (2). The use of antibiotics in ruminant diets during breeding seasons increases reproductive performance, optimizes forage utilization, and leads to quantifiable benefits (3). However, the excessive use of antibiotics in regular animal feed has resulted in various adverse consequences, such as residues in meat and dairy products and the development of resistance to antimicrobial agents. Recent studies have reported that antimicrobial-resistant bacteria are widespread in animal husbandry, with numerous antibiotic-resistant genes identified within the isolated bacteria (4, 5). These concerns have prompted researchers to explore new alternative feed additives for animal husbandry. Phytogenic feed additives have been widely used to improve the reproductive efficiency of domesticated animals (6). Sultanayeva et al. demonstrated that the dietary administration of balsamic poplar buds enhanced goat milk production by 34.2% without compromising milk quality (7). In post-partum anestrus goats, administering two injections of PGF2α in conjunction with microalgae supplementation resulted in a significant increase in serum estradiol 17β levels and a decrease in serum malondialdehyde (8). Ardani et al. recently reported that substituting high-protein concentrates with a ration comprising 30% *Indigofera zollingeriana* and 1% *Uncaria 17gambir* significantly improves nutrient digestibility, enhances mile yield, and reduces methane gas production in Etawa Crossbreed goats (9). Additionally, replacing the company’s forages with phytogenic mixtures, which consist of *Mirasolia diversifolia*, *Gliricidia sepium*, *Indigofera zoolingeriana*, and *palm concentrate*, successfully sustains the intake, digestive efficiency, dairy output, and milk quality produced by Etawa crossbreed dairy goats (10). In male lambs exposed to heat stress, feeding a dietary cation-anion difference can effectively stabilize their production and digestibility without adversely affecting rumen fermentation (11). Owing to their antioxidant and anti-inflammatory effects, as well as their low toxicity, there is a growing trend toward utilizing phytogenics to enhance animal production (12).

Herba epimedii (*H. epimedii*), a traditional Chinese medicines, has long been used for its ability to nourish yin and the kidney, as well as to strengthen the musculoskeletal system (13). For centuries, the *H. epimedii* has been employed in Chinese herbal medicine to enhance male potency (14). Icariin (ICA), the primary flavonoid glucoside derived from *H. epimedii*, is recognized for its diverse pharmacological properties, including anti-inflammatory, antioxidant, estrogenic-like activity, immune regulation, and improvements in reproductive endocrinology (15). According to traditional Chinese medicine principles, kidney yang deficiency syndrome (KYDS) is a metabolic disorder resulting from neuroendocrine dysfunction. The primary clinical manifestations in male patients with KYDS include infertility, decreased libido, reduced sperm motility, and nocturia (16). Previous research has shown that impairment of the hypothalamic–pituitary-gonadal axis is responsible for KYDS, while functional abnormalities in the gonads, thyroid, and adrenal glands are major pathological phenomena in KYDS (17). ICA administration significantly improves sexual function and upregulates the expression of gonadal androgen receptor genes in KYDS mice (18). Moreover, ICA exhibits testosterone-mimetic properties and has the potential to improve erectile function in aged male rats and streptozotocin-induced diabetic rats (19). Additionally, ICA administration has the potential to restore proliferating cell nuclear antigen (PCNA) expression and facilitate germ cell proliferation in high-fat diet rats (20).

Given its observed positive impact on the male reproductive system, it is reasonable to hypothesize that the inclusion of ICA as a feed additive in ruminant diets could potentially improve reproductive performance during the breeding season. While previous studies have primarily focused on the pharmacological effects of ICA on human and mouse reproductive systems, there is a lack of research exploring its potential applications in animal husbandry. Consequently, our study aims to evaluate the influence of ICA on serum hormone levels, semen quality, and spermatogenesis in male dairy goats.

## Materials and methods

### Animal experiments and samples collection

Guanzhong dairy goats are seasonal breeders, mating from August to December (breeding season) and remaining reproductively dormant from January to July (non-breeding season) (21). In the present study, twelve healthy adult male Guanzhong dairy goats, aged 18–24 months with a body weight of 35.7 ± 5.14 kg, were used during the breeding season. The dairy goats were randomly divided into two groups of six each based on body weight. Given that the spermatogenesis cycle and sperm transit through the epididymis in animals usually last about 70–80 days, the experimental group of dairy goats was administered ICA orally at a dosage of 50 mg/kg/day for a consecutive period of 80 days. The weight of the goats was recorded weekly, and the ICA dosage was adjusted accordingly.

### Collection of semen and blood samples

Samples from the dairy goats were collected at specified intervals on days 0, 10, 20, 40, 60, and 80. The ejaculate volume of each goat was recorded, and the collected semen samples were immediately used for quality analysis using the CASA system. To examine serum hormone levels, blood samples were collected, and the serum was stored at −80°C. At the end of the experiment, all goats were euthanized by intravenous administration of pentobarbital sodium. Testes and epididymides were immediately collected and carefully prepared for weighing on an electronic balance.

### Enzyme-linked immunosorbent assay

The serum samples were centrifuged at 3,000 rpm for 15 min, and the supernatants were collected. The serum levels of GnRH (gonadotropin-releasing hormone), GnIH (gonadotropin-inhibiting hormone), LH (luteinizing hormone), FSH (follicle-stimulating hormone), and testosterone were quantified using goat ELISA kits supplied by Shanghai Enzyme Link Biotechnology Co., Ltd., China, according to manufacturer’s instructions.

### Concentration of dopamine (DA) and 5-hydroxytryptamine (5-HT) in the hypothalamus of dairy goats

After the euthanasia of the dairy goats, tissue samples from the hypothalamus were promptly and meticulously collected and then stored at −80°C. The samples were homogenized in a solution of ice-cold PBS. After centrifugation at 3500 rpm for 10 min, the supernatant was used to quantify DA and 5-HT levels. The experimental procedures for determining DA and 5-HT levels followed the protocols of Nanjing Jiancheng Bioengineering Institute, China (22).

### Measurement of CAT, T-SOD, GSH-PX, T-AOC, and MDA levels

Testis samples were added to a pre-cooled 0.9% sodium chloride solution at a weight/volume ratio of 1:9 and then homogenized on ice. The homogenate was centrifuged at 3000 rpm for 10 min at 4°C. The testicular supernatant obtained at 80 days and serum samples collected at different time points were used to determine the concentrations of catalase (CAT), total superoxide dismutase (T-SOD), glutathione peroxidase (GSH-PX), total antioxidant capacity (T-AOC), and malondialdehyde (MDA) using protocols from Nanjing Jiancheng Bioengineering Institute, China.

### Sperm kinematics analysis by CASA system

Semen samples from dairy goats were obtained at the indicated time points (0, 10, 20, 40, 60, and 80 days) using an artificial vagina. Semen volume was quantified, and color observation was performed. Sperm kinematics were assessed using a computer-assisted semen analysis system (CASA). The semen samples were diluted with sterilized, pre-warmed sodium saline solution to achieve a concentration of 2 × 10^7^ sperm/mL. A drop of diluted sperm suspension was placed on an ordinary microscope slide and covered with a coverslip. Subsequently, a pre-warmed Makler counting chamber was positioned on the phase-contrast microscope (Nikon, Japan), and the analysis involved examining successive digitized images taken from a single field at a 200× magnification. For each sample, more than five fields per drop were analyzed by evaluating at least 500 sperm cells. Regarding the setting parameters for the analysis, sperm with a VAP (average path velocity) of more than 15 μm/s were classified as motile.

### Organ coefficients of the testes and epididymides

At the end of the experiment, the weight of testes and epididymides were measured using an electronic balance. The organ coefficients were then calculated using the formula: organ coefficient = organ weight/body weight (g/kg) (23).

### Light microscopy

After fixing the testes and epididymides samples from dairy goats in a 4% paraformaldehyde solution at room temperature for 24 h, the samples were embedded in paraffin. The samples were then cut into 5 μm thick slices and subjected to hematoxylin and eosin (H&E) staining for histological examination using light microscopy (LEICA DM6 B, Germany).

### Immunohistochemistry (IHC)

For immunohistochemistry analysis, sample sections were deparaffinized and rehydrated as previously described (21). The sections were then treated with a 3% H_2_O_2_ solution for 10 min to remove any endogenous peroxidase activity. After three washes with PBS, the sections were boiled in sodium citrate buffer for 10 min to retrieve antigens. The sections were then treated with 5% bovine serum albumin (BSA) for 30 min to reduce nonspecific binding, followed by overnight incubation at 4°C with primary antibodies: Anti-3β-HSD antibody (1:100, A1823, ABclonal); Anti-FSHR antibody (1:100, AF5242, Affinity); Anti-LHR antibody (1:100, AF9140, Affinity); Anti-AR antibody (1,100, 66,747-1-Ig, Proteintech). After rinsing with PBS, the sections were exposed to the secondary antibody for 30 min at 37°C. Finally, the sections were washed three times with PBS and positive reactions were observed using a DAB kit.

### Confocal microscopy

After antigens retrieval, testis sections were immersed in 5% BSA for 30 min to reduce nonspecific binding. The sections were then incubated overnight at 4°C with primary antibodies: Anti-PGP 9.5 antibody (1:200, BF8240, Affinity); Anti-DDX4 antibody (1:200, A15624, ABclnoal); Anti-c-kit antibody (1:200, AF6153, Affinity); Anti-SCP3 antibody (1:200, ab97672, Abcam). After three washes with PBS, the appropriate fluorescent secondary antibody was applied and incubated for 1 h at 37°C. The sections were then stained with DAPI to visualize the cell nucleus and positive reactions were observed using confocal laser scanning microscopy (LEICA TCS SP8, Germany).

### qPCR

Total RNA was extracted from the testes of dairy goat using the Trizol reagent kit (Thermo Fisher Scientific, Inc., USA). A Thermo Scientific NanoDrop 2000 spectrophotometer was used to determine the concentration of total RNA. Subsequently, cDNA was synthesized by reverse transcription of the RNA samples using the PrimeScript reverse transcription reagent kit (TransGen Biotech, Beijing, China). Quantitative PCR analyses were performed using the SYBR Green assay system on the Applied Biosystems 7,500 Real-Time PCR System (Applied Biosystems, Foster City, 273 CA, USA) (20). A final volume of 10 μL per reaction was prepared with 0.5 μL of cDNA as a template. To ensure accurate, each samples was subjected to triplicate reactions. The mRNA expression levels were estimated using the delta–delta Ct (ΔΔCt) method, with *β-actin* serving as the internal control. The primers used in this study are listed in Supplementary Table S1.

### Western blotting

The testis samples of dairy goats were washed three times with ice-cold PBS and then homogenized in RIPA lysis buffer containing protease inhibitors. After incubation on ice for 30 min, the samples were centrifuged at 12,000 rpm for 15 min. The protein concentrations of the supernatants were determined using a BCA kit. Equal amounts of protein were separated by 10% SDS-PAGE gels and transferred to a PVDF membrane. The membranes were incubated with 5% non-fat milk in Tris-buffered saline containing 0.05% Tween-20 (TBST) for 45 min at room temperature. Thereafter, the membranes were incubated overnight at 4°C with primary antibodies, including AR (1:1000), LHR (1:1000), 3*β*-HSD (1:1000), StAR (1:1000, 12,225-1-AP, Proteintech), PGP 9.5 (1:800), DDX4 (1:800), SCP3 (1:1000), SOX9 (1:1000, A19710, ABclonal) and β-Actin (1:1500, ab8227, Abcam). The membranes were then incubated with the appropriate secondary antibodies for 30 min at 37°C. Finally, the Bio-Rad ChemiDoc system was used to analyze protein band intensities. The acquired data were statistically analyzed using Image J software, with *β*-actin serving as an internal control to normalize protein loading.

### Statistical analysis

Data in present study were reported as means ± SEM. Statistical analysis was performed using GraphPad Prism 8.0 software. The normality of the data was assessed using the Shapiro Wilk normality test. A two-tailed Student’s T test was employed to determine statistical difference between two groups. One-way ANOVA and Tukey’s multiple comparisons test were used to compare multiple groups. Differences were considered significant at *p* < 0.05 (*) or *p* < 0.01 (**).

## Results

### Effect of ICA on serum hormones and antioxidant capacity of dairy goats

**Figure 1:**
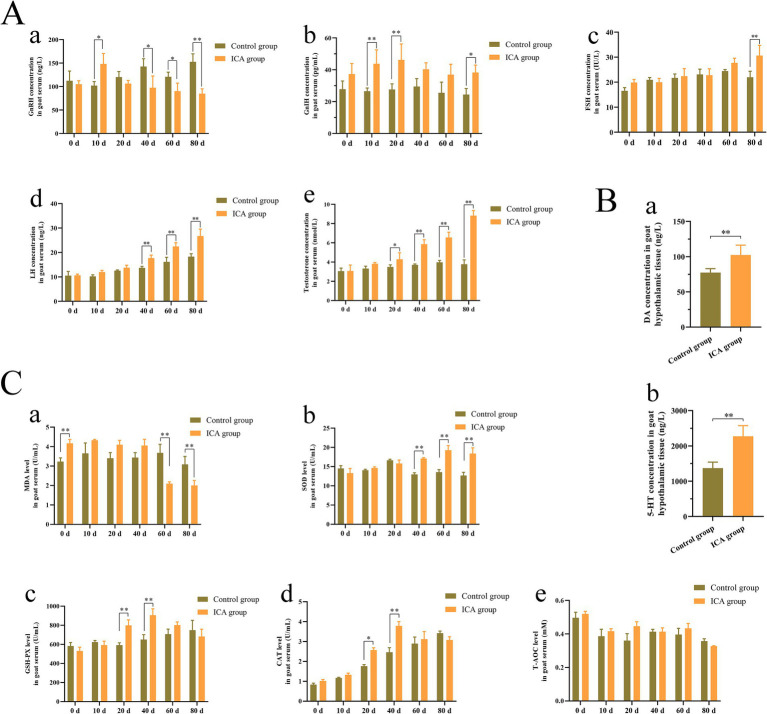
Administration of ICA to dairy goats increased serum hormone levels and enhanced antioxidant capacity. (A) The serum hormone levels were examined with an ELISA kit. (a) GnRH. (b) GnIH. (c) FSH. (d) LH. (e) Testosterone. (B) The hypothalamic DA and 5-HT concentrations were examined by commercial kits. (a) DA. (b) 5-HT. (C) The antioxidant enzyme levels were examined by commercial kits. (a) MDA. (b) SOD. (c) GSH-PX. (d) CAT. (e) T-AOC. Each value represents the mean ± SEM, *p < 0.05, **p < 0.01.

As shown in Figure 1, the serum concentration of GnRH initially increased, followed by a subsequent decrease after ICA administration (Figure 1Aa). Compared with the control group, the serum GnIH concentration showed an upward trend after ICA treatment (Figures 1A,B). The serum levels of FSH, LH, and testosterone progressively increased in a time-dependent manner (Figures 1Ac,d,e). Hypothalamic DA and 5-HT levels exhibited a significant increase in the ICA group compared to the control group (Figures 1Ba,b). In addition, there were significant differences in serum T-AOC, CAT, GSH-PX, SOD, and MDA levels between the groups. MDA levels were significantly downregulated after 60 days of administration, while SOD activity gradually increased over time and showed a significant increase after 40 days of treatment (Figures 1Ca,b). Serum GSH-PX and CAT levels were significantly higher in the ICA group on both day 20 and day 40 compared to controls (Figures 1Cc,d). However, no significant difference was observed for the T-AOC level among the groups (Figure 1Ce).

### Effect of ICA on semen quality of dairy goats

**Figure 2:**
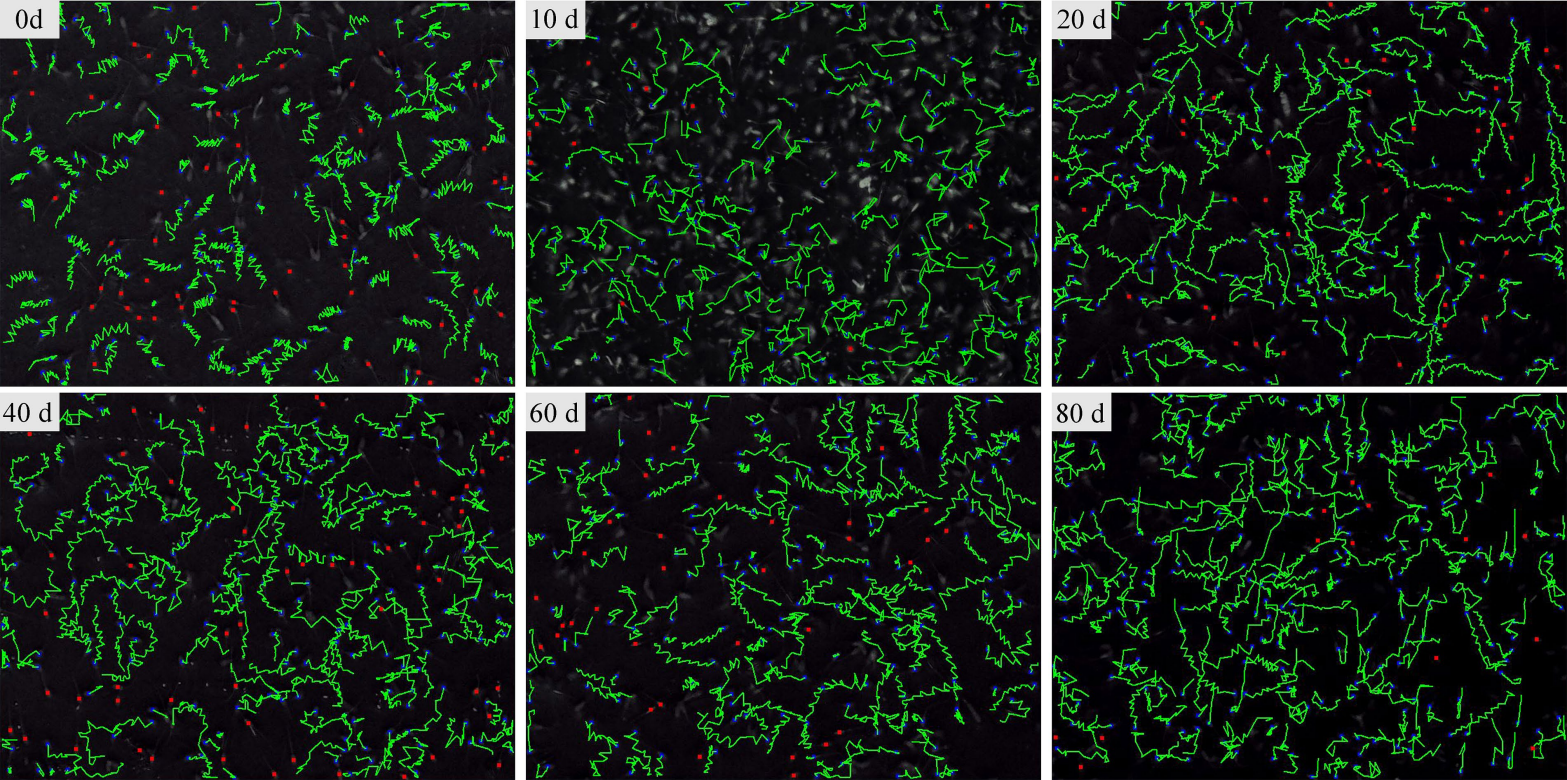
The sperm movement trajectory of dairy goats was analyzed using the CASA system. Sperm movement trajectory was recorded on days 0, 10, 20, 40, 60, and 80 after the administration of ICA.

The CASA system was used to assess sperm motility parameters at different time points during ICA administration. Sperm with strong forward motion exhibited oscillatory movements in their tail flagellum (Figure 2). The CASA system documented sperm movement trajectories and showed a gradual increase in the percentage of total motile and progressively motile sperm with the extension of ICA treatment (Figure 2). As shown in Table 1, daily oral administration of ICA resulted in an upward trend in sperm quality, characterized by increased ejaculate volume (EV), elevated total motility sperm (TM), and higher linearity (LIN). Furthermore, sperm kinematics such as straight line velocity (VSL), curvilinear velocity (VCL), and average path velocity (VAP) also exhibited an increasing trend with the prolongation of ICA treatment.

**Table 1:** Sperm motility and kinematics of dairy goats were analyzed using CASA system.

Items	Control group	ICA group				
		10d	20d	40d	60d	80d
EV (mL)	0.99 ± 0.19	1.00 ± 0.15	1.21 ± 0.26	1.23 ± 0.23	1.31 ± 0.35	1.26 ± 0.29
TM (%)	76.64 ± 5.97^a^	78.70 ± 5.70^a,b^	81.25 ± 5.38^a,b^	81.78 ± 5.98^a,b^	88.80 ± 2.85^a,b^	93.33 ± 2.33^b^
VSL (μm/s)	22.26 ± 1.89	25.93 ± 1.30	31.92 ± 4.09	32.44 ± 5.14	32.72 ± 3.21	30.67 ± 1.04
VCL (μm/s)	58.85 ± 6.55	53.85 ± 1.67	58.76 ± 3.99	66.76 ± 5.16	67.56 ± 2.95	63.82 ± 6.76
VAP (μm/s)	41.88 ± 2.49	39.21 ± 5.25	41.80 ± 1.52	47.66 ± 2.35	49.59 ± 6.55	42.90 ± 3.31
ALH (μm)	19.65 ± 3.84	16.31 ± 1.35	17.29 ± 0.95	20.94 ± 3.43	20.53 ± 3.74	17.37 ± 2.50
WOB	0.92 ± 0.04	0.91 ± 0.02	0.88 ± 0.03	0.93 ± 0.05	0.94 ± 0.04	0.90 ± 0.07
BCF	0.76 ± 0.06	0.76 ± 0.04	0.74 ± 0.09	0.76 ± 0.07	0.72 ± 0.07	0.74 ± 0.06
LIN	0.37 ± 0.03^a^	0.47 ± 0.05^a,b^	0.53 ± 0.04^b^	0.47 ± 0.06^a,b^	0.47 ± 0.05^a,b^	0.53 ± 0.05^b^
MAD	317.64 ± 20.89^a^	258.64 ± 11.98^b^	261.15 ± 11.18^b^	263.81 ± 21.88^b^	233.12 ± 12.65^b^	255.81 ± 10.11^b^
STR	0.58 ± 0.04	0.65 ± 0.07	0.73 ± 0.04	0.65 ± 0.05	0.66 ± 0.07	0.74 ± 0.09

EV, ejaculate volume; TM, total motile sperm; VSL, straight line velocity; VCL, curvilinear velocity; VAP, average path velocity; ALH, amplitude of lateral head displacement; WOB, wobble; BCF, beat cross frequency; LIN, linearity; MAD, mean angular displacement; STR, straightness. The values are presented with mean SEM and values within a row with different superscripts (a, b) indicate significant differences (*p* < 0.05).

### Effect of ICA on the morphology of goat testes and epididymides

**Figure 3:**
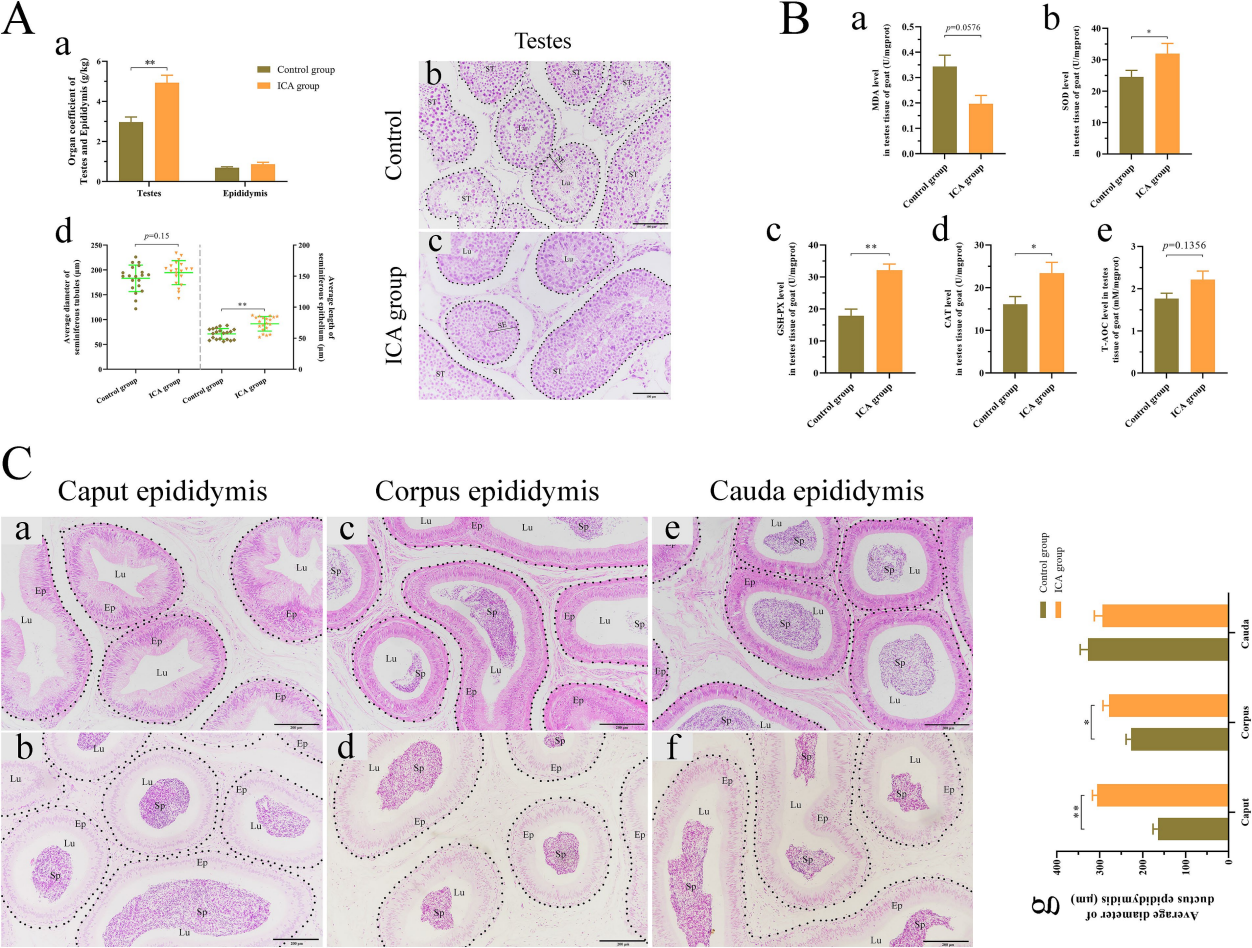
Administration of ICA to dairy goats enhanced testicular spermatogenesis and antioxidant capacity. (A) Effect of ICA on spermatogenesis. (a) The organ coefficient of testes and epididymides. (b) H&E staining of testes in the control group. (c) H&E staining of testes in ICA group. ST, seminiferous tubules; SE, seminiferous epithelium; Lu, lumen; Scale bar: b and c = 100 μm. (d) The average diameter of ST and the average length of SE based on H&E figures. (B) Effect of ICA on testicular antioxidant capacity. (a) MDA. (b) SOD. (c) GSH-PX. (d) CAT. (e) T-AOC. (C) Effect of ICA on epididymal sperm. (a) The caput epididymis of the control group. (b) The caput epididymis of the ICA group. (c) The corpus epididymis of the control group. (d) The corpus epididymis of the ICA group. (e) The cauda epididymis of the control group. (f) The cauda epididymis of the ICA group. (g) The histogram represents the quantification of the average diameter of ductus epididymis. Ep: epithelium; Sp: spermatozoa; Lu: lumen; Scale bar = 200 μm. Each value represents the mean ± SEM, *p < 0.05, **p < 0.01.

Our results, illustrated in Figure 3Aa, indicated that ICA treatment significantly elevated the testes coefficients but had no impact on the epididymal coefficient compared to the control group. H&E staining showed that both Leydig cells and Sertoli cells retained their normal morphologies, a continuous and intact basement membrane was present in the seminiferous tubules, and germ cells were arranged in a regular pattern in both groups (Figures 3A,Bc). The average length of the seminiferous epithelium was significantly greater in the ICA group than in the control group (Figure 3Ad). Additionally, we analyzed the antioxidant capacity of the testes and found a significant increase in SOD, GSH-PX, and CAT levels in the ICA group compared to the control group (Figure 3B). Furthermore, H&E staining revealed that sperm in the control group were primarily located in the corpus and cauda epididymis (Figure 3C). However, in the ICA group, sperm were also observed in the caput epididymis (Figure 3Cb). Statistical analysis revealed that the average diameter of the ductus epididymidis at the caput and corpus was larger in the ICA group than in the control group (Figure 3Cg).

### Effect of ICA on testicular spermatogenesis and steroidogenesis

**Figure 4:**
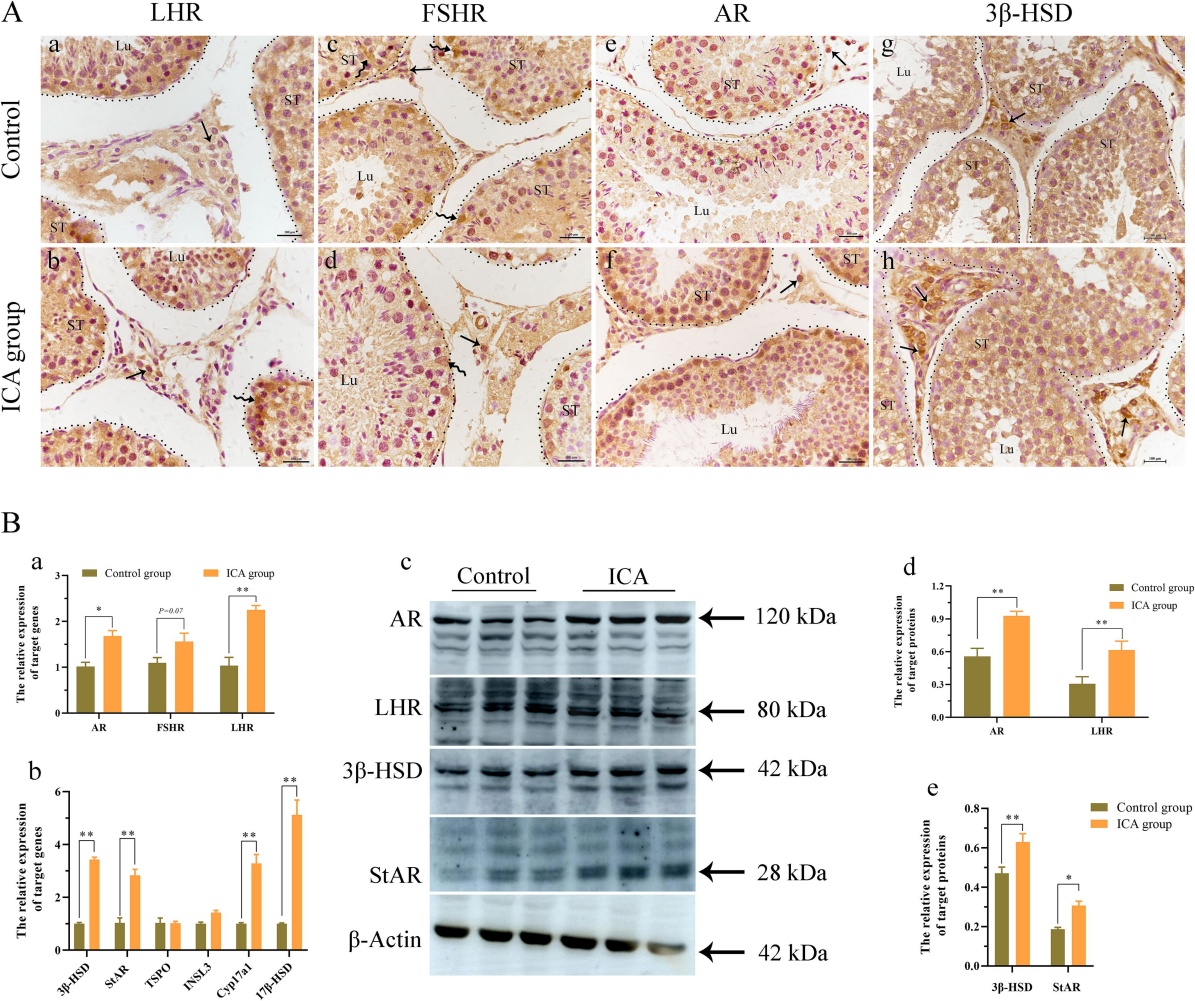
Administration of ICA to dairy goats increased the expression of hormone receptors and testosterone synthesis in the testes. (A) Immunohistochemistry analyzes the distribution of hormonal receptors in the testes. (a) The distribution of LHR in the control group. (b) The distribution of LHR in the ICA group. (c) The distribution of FSHR in the control group. (d) The distribution of FSHR in the ICA group. (e) The distribution of AR in the control group. (f) The distribution of AR in the ICA group. (g) The distribution of 3*β*-HSD in the control group. (h) The distribution of 3β-HSD in the ICA group. ST, seminiferous tubules; Lu, lumen; Curved arrow, Sertoli cells; Arrow, Leydig cells; Scale bar = 100 μm. (B) qPCR and western blot analyze the mRNA and protein expression in the testes. (a) The mRNA levels of *AR*, *LHR*, and *FSHR* were examined by qPCR. (b) The mRNA levels of steroidogenesis enzymes were examined by qPCR. (c) Western blot analysis was performed to measure the expression of target proteins. β-actin was utilized as a loading control. (d,e) The histogram represents the quantification of proteins levels. Each value represents the mean ± SEM, *p < 0.05, **p < 0.01.

The distribution and expression of LHR, FSHR, and AR in the testes were examined. IHC analysis revealed that LHR was predominantly localized in Leydig cells and Sertoli cells (Figures 4Aa,b). The majority of FSHR expression was observed in Sertoli cells (Figures 4Ac,d), while AR expression was detected in Leydig cells, Sertoli cells, and germ cells (Figures 4Aa–f). There was a significant increase in the mRNA levels of AR, FSHR, and LHR in the ICA group compared to the control group (Figure 4Ba). Consistent with the mRNA levels, the protein levels of AR and LHR were significantly increased in the ICA group compared to the control group (*p* < 0.01) (Figures 4Bc,d). In addition, we analyzed the increased mRNA and protein levels of enzymes responsible for testosterone synthesis in the ICA group (Figures 4Bb,c,e).

**Figure 5:**
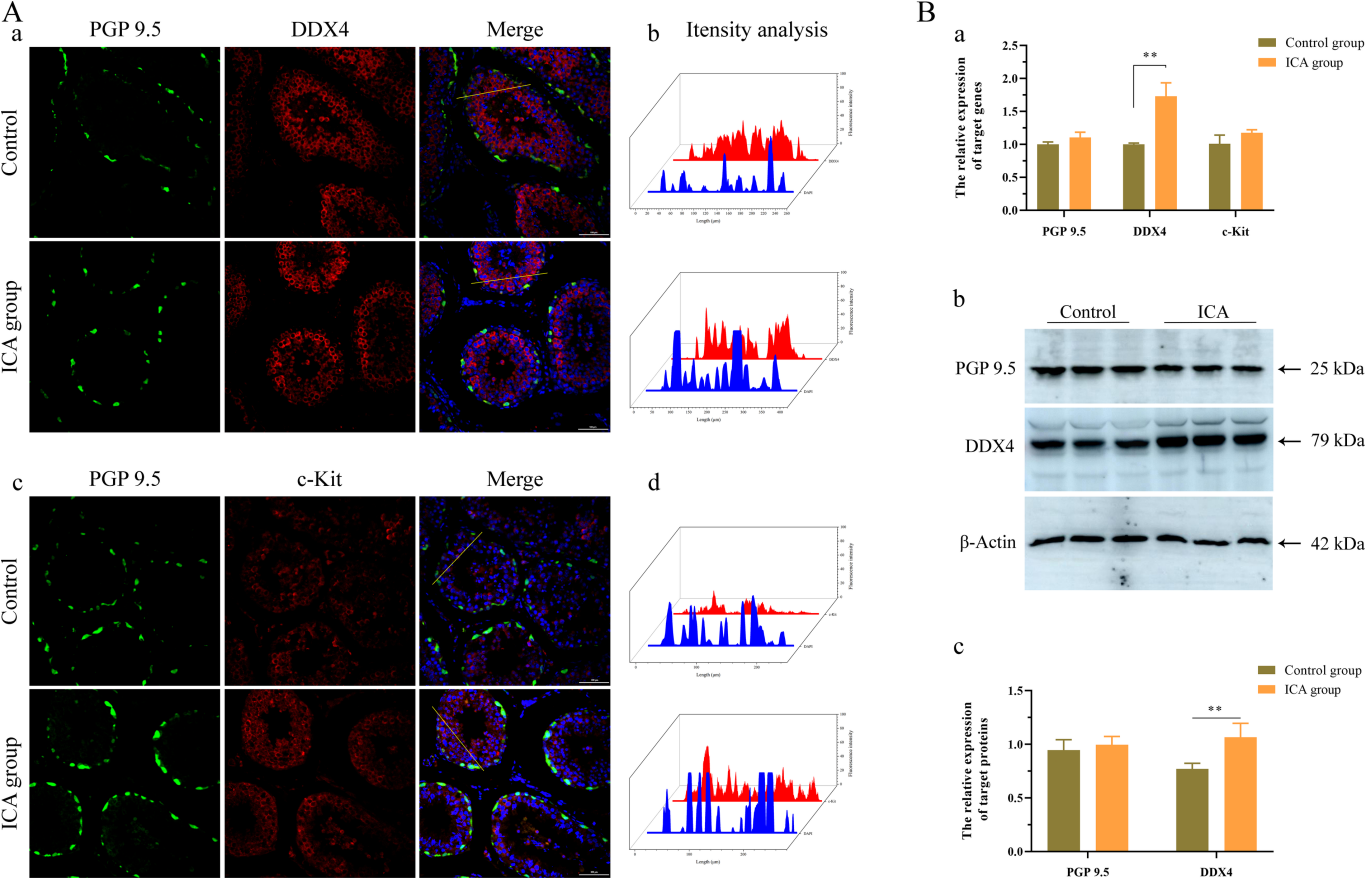
Effect of ICA administration on male germ cell markers of dairy goats. (A) Confocal microscopy analyzes the distribution of PGP9.5, DDX4, and c-Kit in the testes. (a) Immunofluorescence analysis showed the expression of PGP9.5 and DDX4. (b) The fluorescence intensity was analyzed on the yellow lines by Fiji software. (c) Immunofluorescence analysis showed the distribution of PGP9.5 and c-Kit. (d) The fluorescence intensity was analyzed on the yellow lines by Fiji software. (B) qPCR and western blot analyze the expression of mRNA and proteins. (a) The mRNA levels of *PGP9.5*, *DDX4*, and *c-Kit* were examined by qPCR. (b) Western blot analysis was performed to measure the expression of target proteins. β-actin was utilized as a loading control. (c) The histogram represents the quantification of proteins levels. Each value represents the mean ± SEM, **p < 0.01.

**Figure 6:**
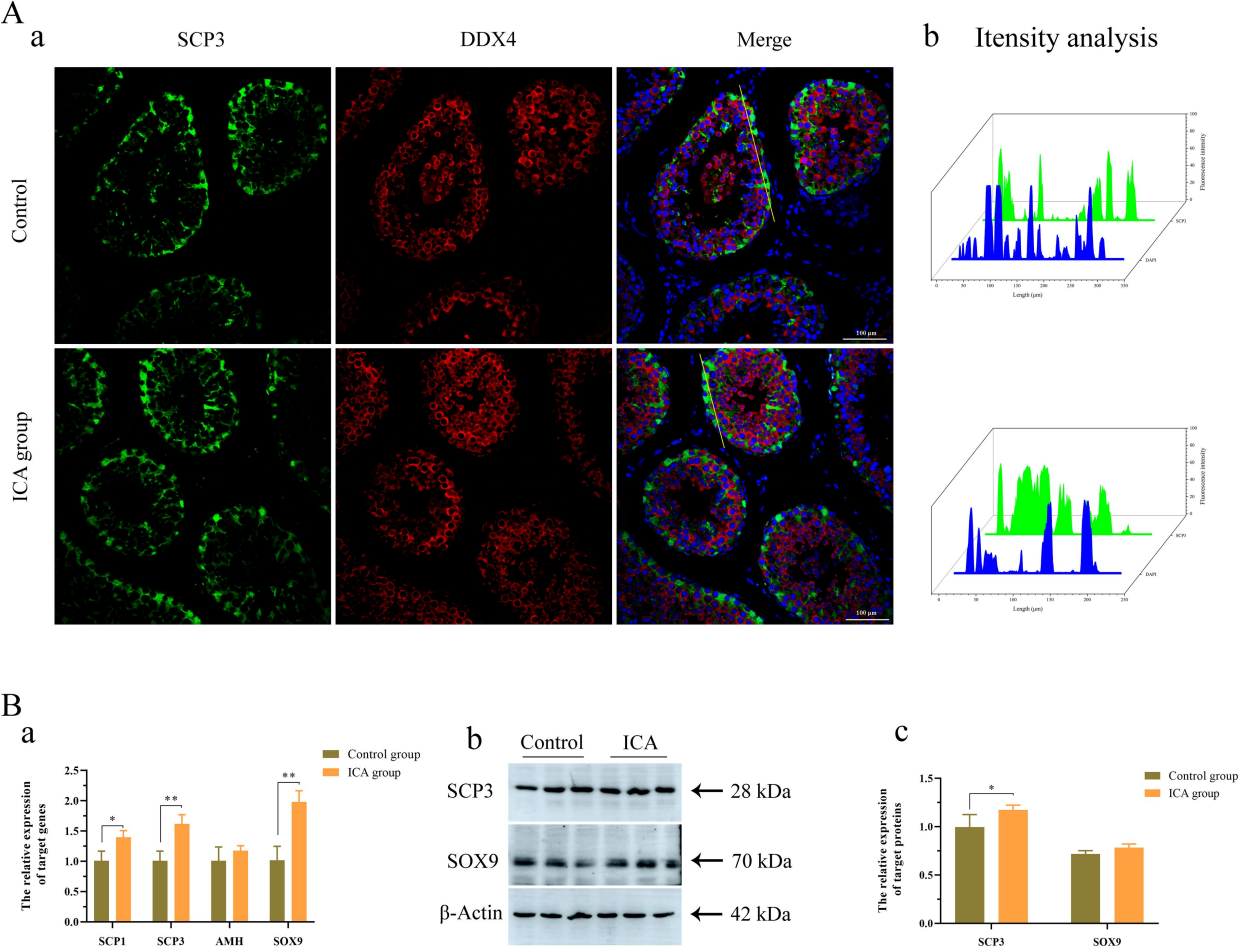
Effect of ICA administration on spermatocytes and Sertoli cells of dairy goats. (A) Confocal microscopy analyzes the distribution of SCP3 and DDX4 in the testes. (a) Immunofluorescence analysis showed the distribution of SCP3 and DDX4. (b) The fluorescence intensity was analyzed on the yellow lines by Fiji software. (B) qPCR and western blot analyze the expression of mRNA and proteins. (a) The mRNA levels of *SCP1*, *SCP3*, *AHM* and *SOX9* were examined by qPCR. (b) Western blot analysis was performed to measure the expression of target proteins. β-actin was utilized as a loading control. (c) The histogram represents the quantification of proteins levels. Each value represents the mean ± SEM, *p < 0.05, **p < 0.01.

Next, we examined the biomarkers of male germ cells in the control and ICA groups. Confocal microscopy analysis revealed that PGP 9.5-positive cells were distributed near the basement membrane of seminiferous tubules (Figure 5Aa). There was no significant difference in the expression level of PGP 9.5 after ICA treatment (Figure 5B). However, DDX4 expression was significantly upregulated in the ICA group compared to the control group, and these DDX4-expressing cells were mainly located on the luminal side (Figures 5Aa,B) c-Kit-positive cells were mainly distributed on the luminal side (Figure 5Ac). We also investigated the distribution and expression patterns of SCP3 in the testes using confocal microscopy and found that it was localized in primary spermatocytes (Figures 6Aa,b). Consistently, both SCP3 mRNA and protein levels were significantly increased in the ICA group compared to the control group (Figures 6Ba–c). SOX9 expression did not change between groups treated with or without ICA (Figure 6B).

## Discussion

Traditional Chinese medicine has a long history of regulating male reproduction function and antioxidant activity. *H. epimedii*, also known as Horny Goat Weed or Yin Yang Huo in Chinese, is a well-known tonic commonly used in traditional Chinese medicine. ICA exhibits a variety of pharmacological and biological activities (24). Treatment with ICA effectively improves spermatogenesis dysfunction and reverses adverse effects by modulating glycolytic activity in obese mice induced by high-fat diets (25). Moreover, oral administration of ICA for 35 consecutive days to normal adult rats significantly increased epididymal sperm counts, testosterone production, and Sertoli cell function (26). In this study, dairy goats were administered ICA orally for 80 consecutive days during the breeding season. After 20 days of treatment, there was a significant increase in ejaculate volume, total sperm motility, and sperm kinematics (Figure 2; Table 1). There were no significant differences in goat body weight between the two groups. At the end of the experiment, ICA treatment increased the organ coefficient of the testes and enhanced spermatogenesis in dairy goats (Figure 3). The regulation of the male reproductive system is primarily orchestrated by the hypothalamic–pituitary-testicular (HPT) axis. Moreover, the age of the animal also influences the gross and histomorphometric characteristics of the pineal gland, and these changes ultimately affect reproductive physiology, especially in seasonally breeding species (27). GnRH released by the hypothalamus stimulates the biosynthesis and secretion of FSH and LH, which then regulate testicular function via their receptors (28). In this study, we observed a significant increase in serum levels of GnRH, LH, and FSH in the ICA group compared to the control group. Consistent with the serum findings, testicular AR and LHR levels were also increased in the ICA group. Hypothalamic secretion of DA and 5-HT is closely linked to male reproductive ability (29). After ICA administration, there was a significant increase in hypothalamic DA and 5-HT levels, suggesting that ICA may influence male reproductive function by modulating the HPT axis. Additionally, in female studies, ICA increases the biosynthesis of oestrogens and progesterone and improves ovarian function (30).

The antioxidant defense system is a protective mechanism that shields organisms from oxidative damage by inhibiting lipid peroxidation and degrading excessive free oxygen radicals. ICA exhibits antioxidant effects that protects tissues and organs from severe oxidative damage (31). The testes are equipped with a complex network of antioxidant enzymes and free radical scavengers to safeguard spermatogenesis and steroidogenesis from oxidative stress (32). The efficiency of the antioxidant defense system gradually decreases with age, leading to the accumulation of ROS in sperm, which further impairs steroidogenesis and spermatogenesis (33). Oral administration of titanium dioxide nanoparticles induces DNA damage, adversely affects epididymal semen quality, and elevates oxidant levels in testicular tissue (34). However, adding spirulina nanoparticles or selenium-coated spirulina nanoparticles to the freezing extender significantly improves the quality of post-thawed bull sperm by enhancing antioxidant biomarkers, reducing lipid peroxidation, and maintaining sperm ultrastructure (35). In the mouse model, ICA attenuates nicotine-induced oxidative damage and reproductive toxicity in the testes (23). Our results demonstrated that ICA treatment upregulated the concentration of antioxidant enzymes, including SOD, GSH-Px, and CAT, in goat serum. However, no alteration in serum T-AOC levels was observed. Evidence from a mouse study suggests that ICA may improve sperm viability by enhancing antioxidant capacity (36). Our study found significantly increased levels of antioxidant enzymes in the testes of the ICA group compared to the control group. Overall, our results are consistent with previous studies in mice and indicate that ICA partially regulates male reproductive function through its antioxidant properties.

Testosterone, a crucial component of sex hormones, plays an indispensable role in stimulating spermatogenesis and maintaining normal sexual behavior. It is well established that ICA exhibits testosterone-like properties and effectively increases circulating testosterone levels of in rat models (19). In this study, the administration of ICA to dairy goats resulted in a significant increase in serum testosterone levels compared to the control group. Steroidogenesis is a complex process that involves multiple enzymes to convert free cholesterol into biologically active steroid hormones. The initial step is the transport of cholesterol from the outer to the inner mitochondria membrane, facilitated by various transport proteins, including StAR, TSPO, and VDAC (37). Our study found that the expression of steroidogenic enzymes was upregulated in the testes after ICA administration compared to the control group. These results support our hypothesis that ICA enhances testosterone synthesis in the testes by regulating steroidogenic enzymes. In prepubertal animals, FSH plays an essential role in initiating the first wave of spermatogenesis. However, in adulthood, spermatogenesis relies primarily on androgens (38). Spermatogenesis is a complex process involving the proliferation, differentiation, and maturation of diploid and undifferentiated spermatogonia into haploid spermatozoa. Identifying stage-specific biomarkers for different germ cells is crucial for studying spermatogenesis and has been extensively investigated (39). PGP9.5 is a deubiquitinase expressed in undifferentiated spermatogonia (40). c-Kit is a cell membrane receptor protein that plays a crucial role in germ cell maturation and serves as a marker for the loss of potency of spermatogonial stem cell until meiosis onset (41). The DDX4 gene encodes an ATP-dependent RNA helicase, present in spermatocytes and spermatids. In postpubertal porcine testes, DDX4 and c-Kit serve as specific markers for differentiated spermatocytes spermatocytes (42). SCP3 is a meiosis-specific structural protein located in the axial and lateral elements of the synaptonemal complex within spermatocytes (43). Our immunofluorescence results showed that PGP9.5-positive cells were located near the basement membrane of the seminiferous tubules, while DDX4- and c-Kit-positive cells were situated on the luminal side. Furthermore, the administration of ICA significantly elevated the testicular expression levels of DDX4 and SCP3, suggesting that ICA may enhance spermatogenesis by increasing sperm production.

## Conclusion

In conclusion, the administration of ICA to dairy goats positively affects male reproductive performance during the breeding season. This is achieved through the modulation of gonadal hormone secretion and the promotion of spermatogenesis, thereby improving male fertility. Additionally, ICA administration enhances the antioxidant defense capacity in dairy goat testes. However, it should be noted that our findings are based on standard animal husbandry practices aimed at investigating the potential effects of phytogenic feed additives. Further research is needed to analyze the regulatory mechanisms of phytogenic feed additives in the male reproductive system.

## Data Availability

The original contributions presented in the study are included in the article/Supplementary material, further inquiries can be directed to the corresponding authors.

## Glossary

ICA: Icariin
CASA: Computer-assisted semen analysis system
GnIH: Gonadotropin-inhibiting hormone
GnRH: Gonadotropin-releasing hormone
LH: Luteinizing hormone
FSH: Follicle-stimulating hormone
DA: Dopamine
5-HT: 5-hydroxytryptamine
KYDS: Kidney yang deficiency syndrome
PCNA: Proliferating cell nuclear antigen
CAT: Catalase
T-SOD: Total- superoxide dismutase
GSH-PX: Glutathione peroxidase
T-AOC: Total-antioxidant capacity
MDA: Malondialdehyde
VAP: Velocity average path
3β-HSD: 3β-hydroxysteroid dehydrogenase
StAR: Steroidogenic acute regulatory protein
TSPO: Translocator protein
VDAC: Voltage-dependent anion channel protein; INSL3: insulin like 3
Cyp17a1: Cytochrome P450 family 17 subfamily A member 1A
17β-HSD: 17β-hydroxysteroid dehydrogenase
AR: Androgen receptor
LHR: Luteinizing hormone receptor
FSHR: Follicle-stimulating hormone receptor
PGP 9.5: Protein gene product 9.5
DDX4: Dead-Box polypeptide 4
c-Kit: Tyrosin-protein kinase kit
CP1: Synaptonemal complex protein 1
CP3: Synaptonemal complex protein 3
AMH: Anti-Mullerian hormone;
SOX9: SRY-box transcription factor 9
HPT: Hypothalamic–pituitary-testes axis
