# Supplementary material for: Effects of icariin as a feed additive on the reproductive function in bucks (Capra hircus)
Source: Front Vet Sci. 2024 Nov 6;11:1467947. doi: 10.3389/fvets.2024.1467947 (PMC11580527; doi:10.3389/fvets.2024.1467947)

**Supplementary File 1.** The corresponding original and uncropped western blot images used in present study.

Marker used in this study (1) AR


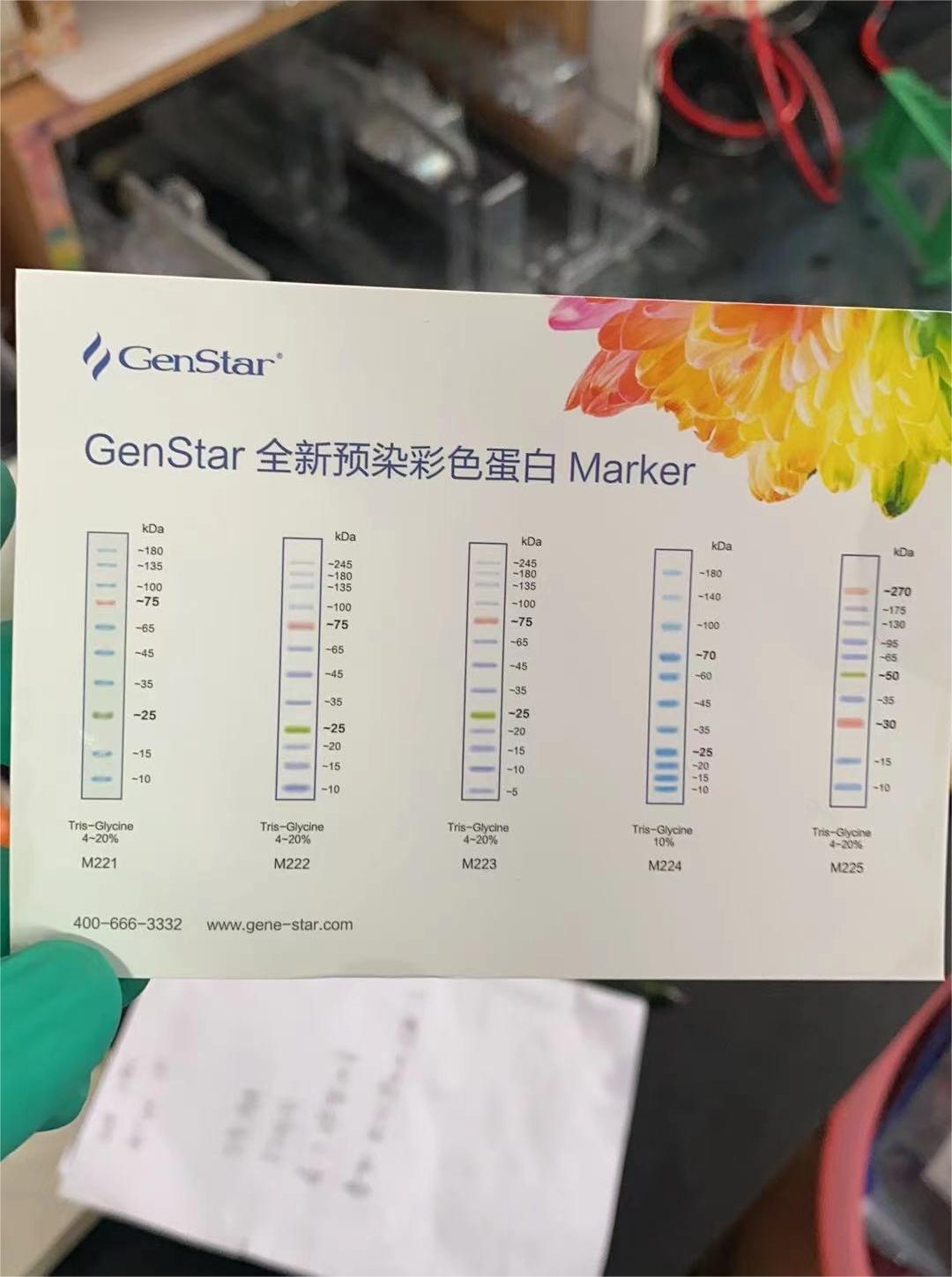

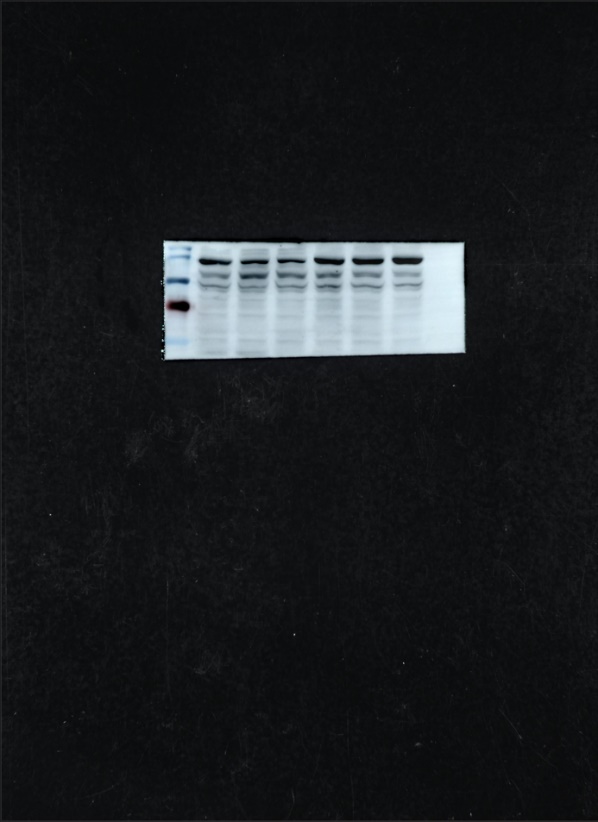


(2) LHR: The band of Marker (75 kDa) affects the expression of target protein, we removed the Marker and re-exposed the target protein.


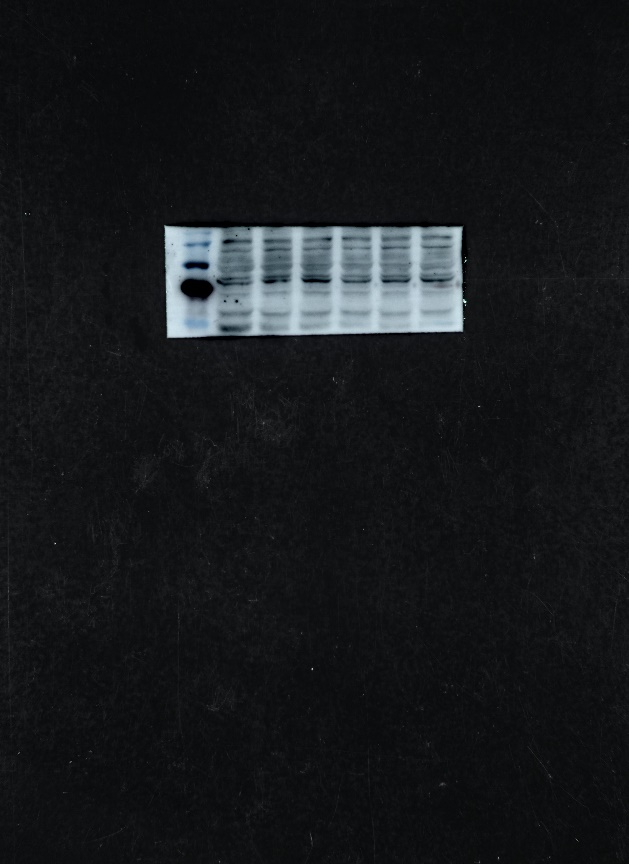

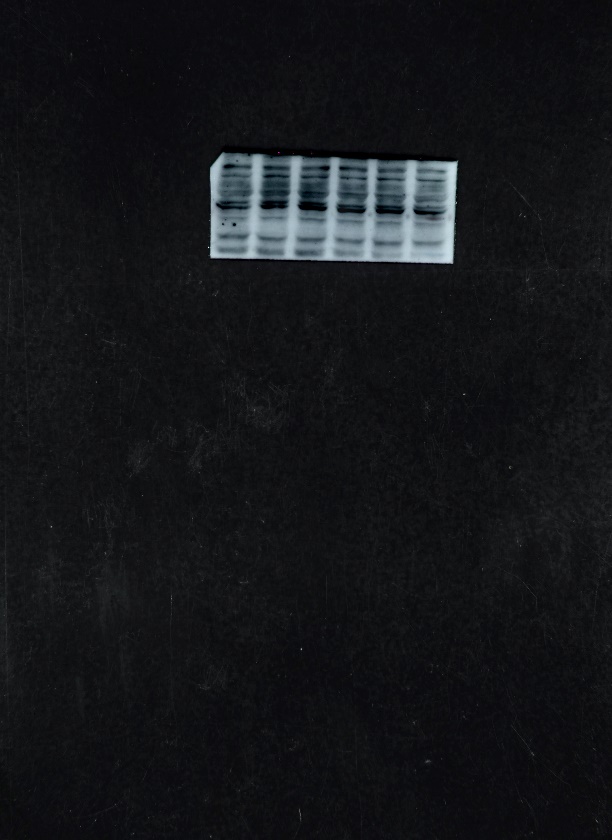


(3) 3β-HSD (4) StAR


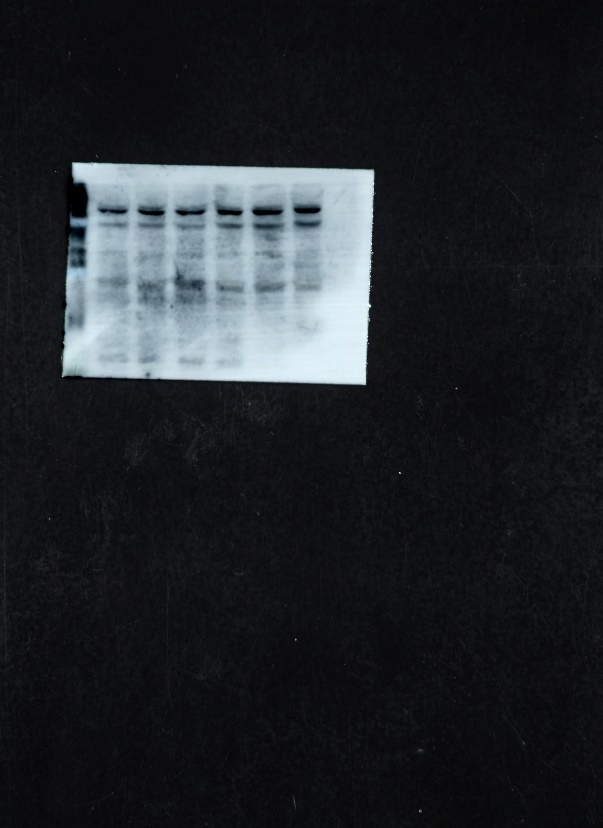

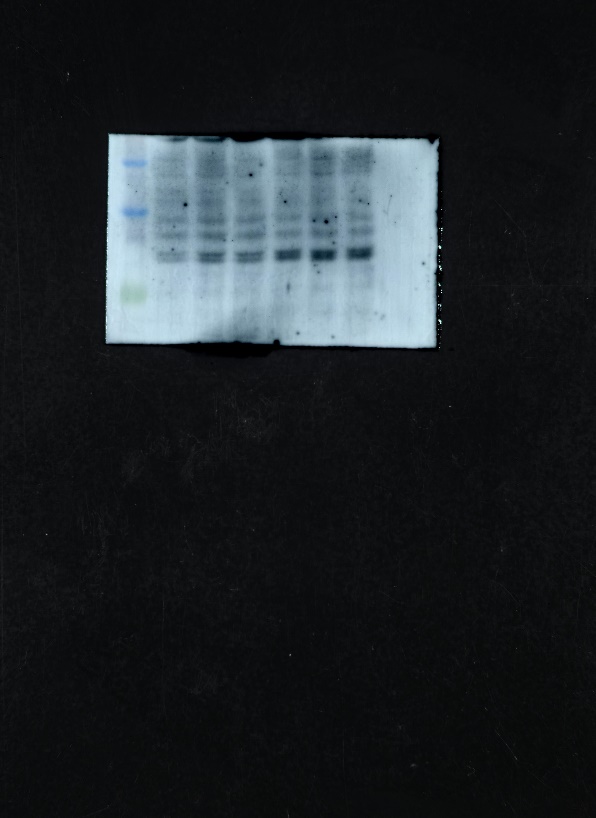


(5) Actin (6) PGP 9.5


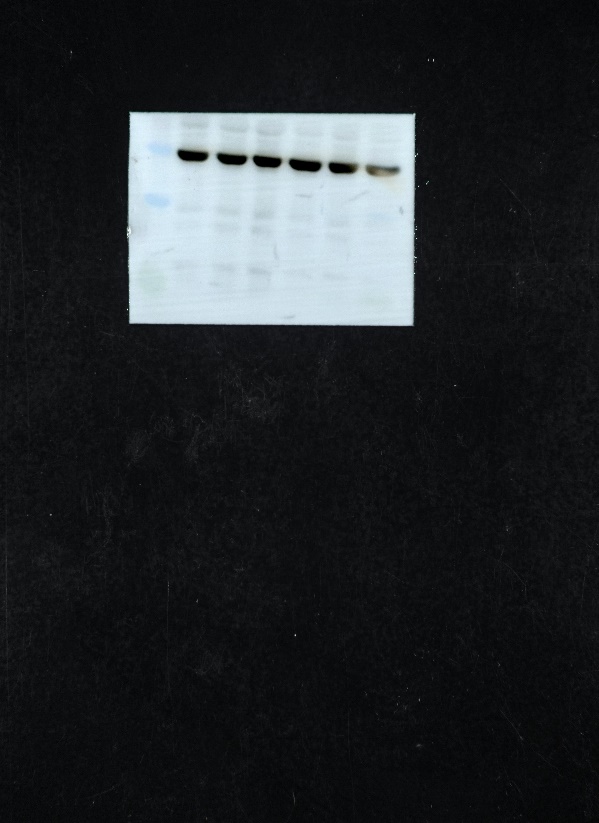

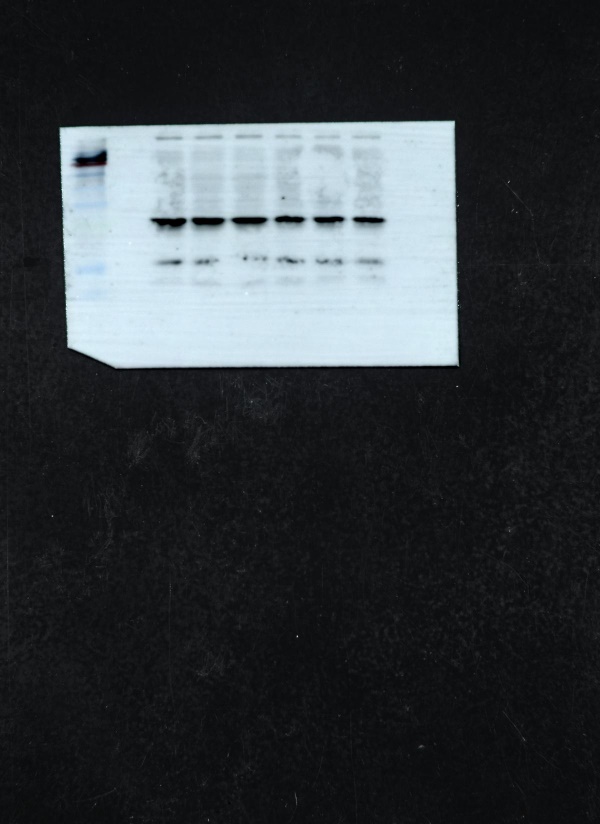


(7) DDX4 (8) Actin


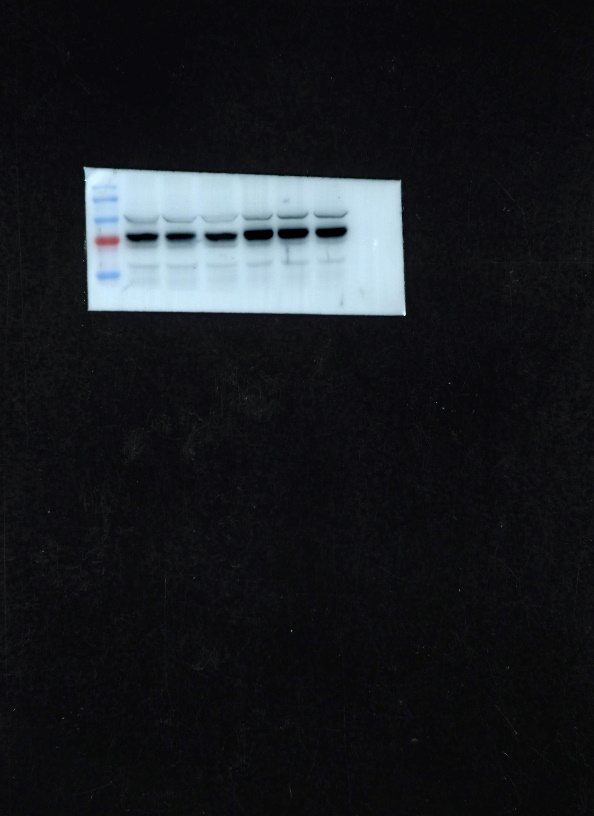

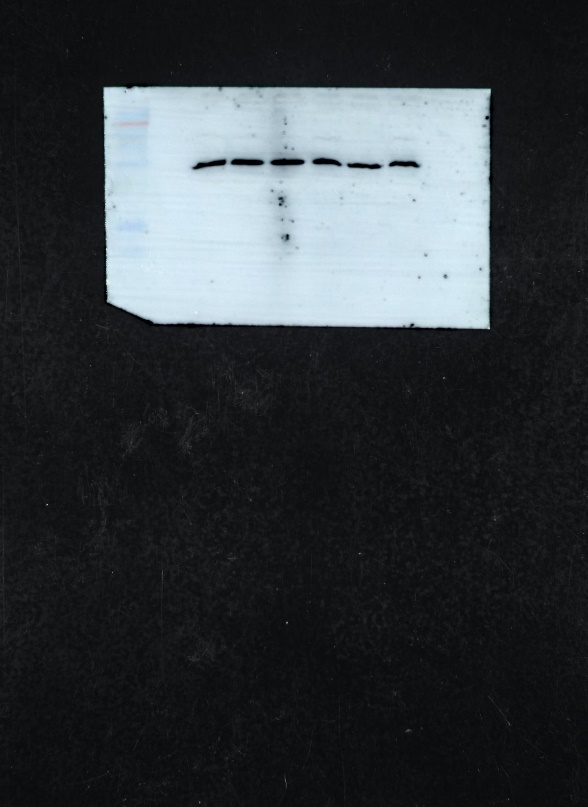


(9) SCP3 (10) SOX9


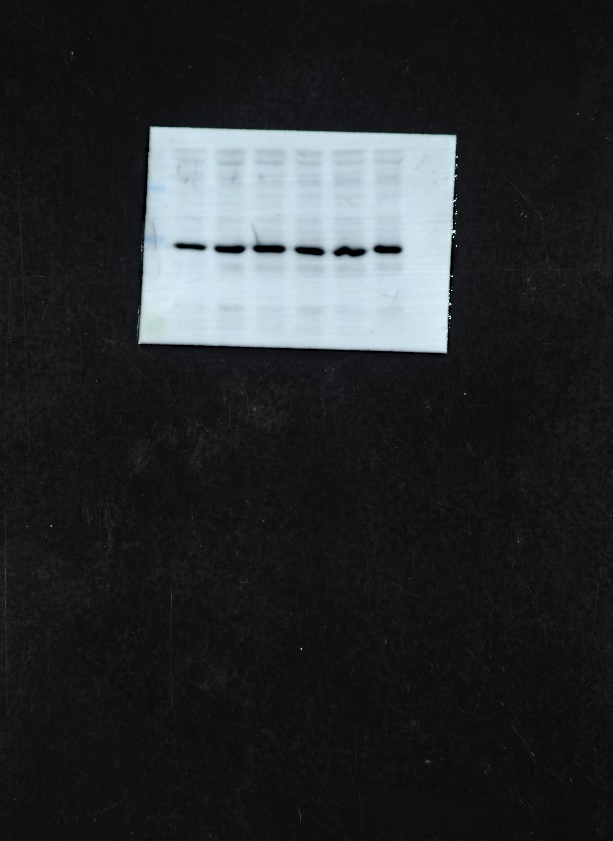

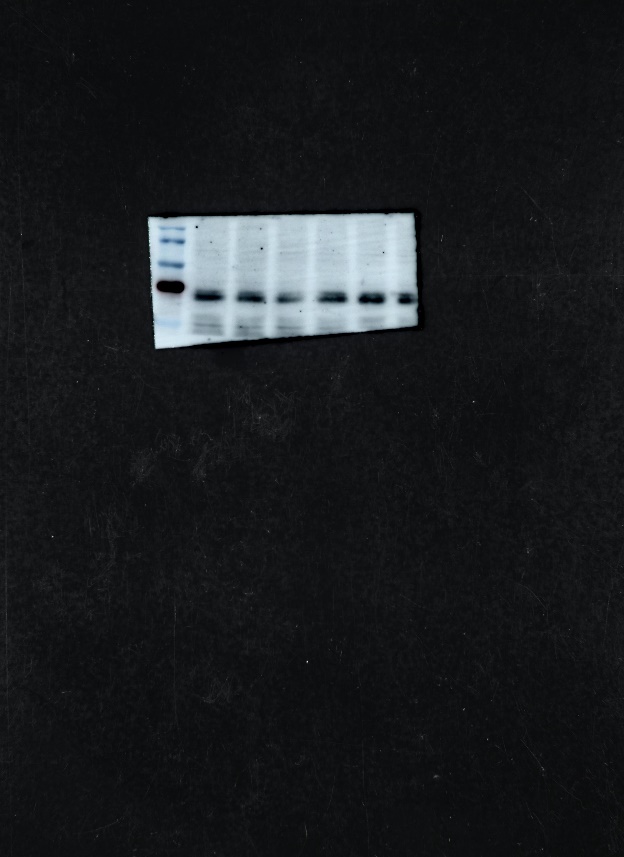


(11) Actin


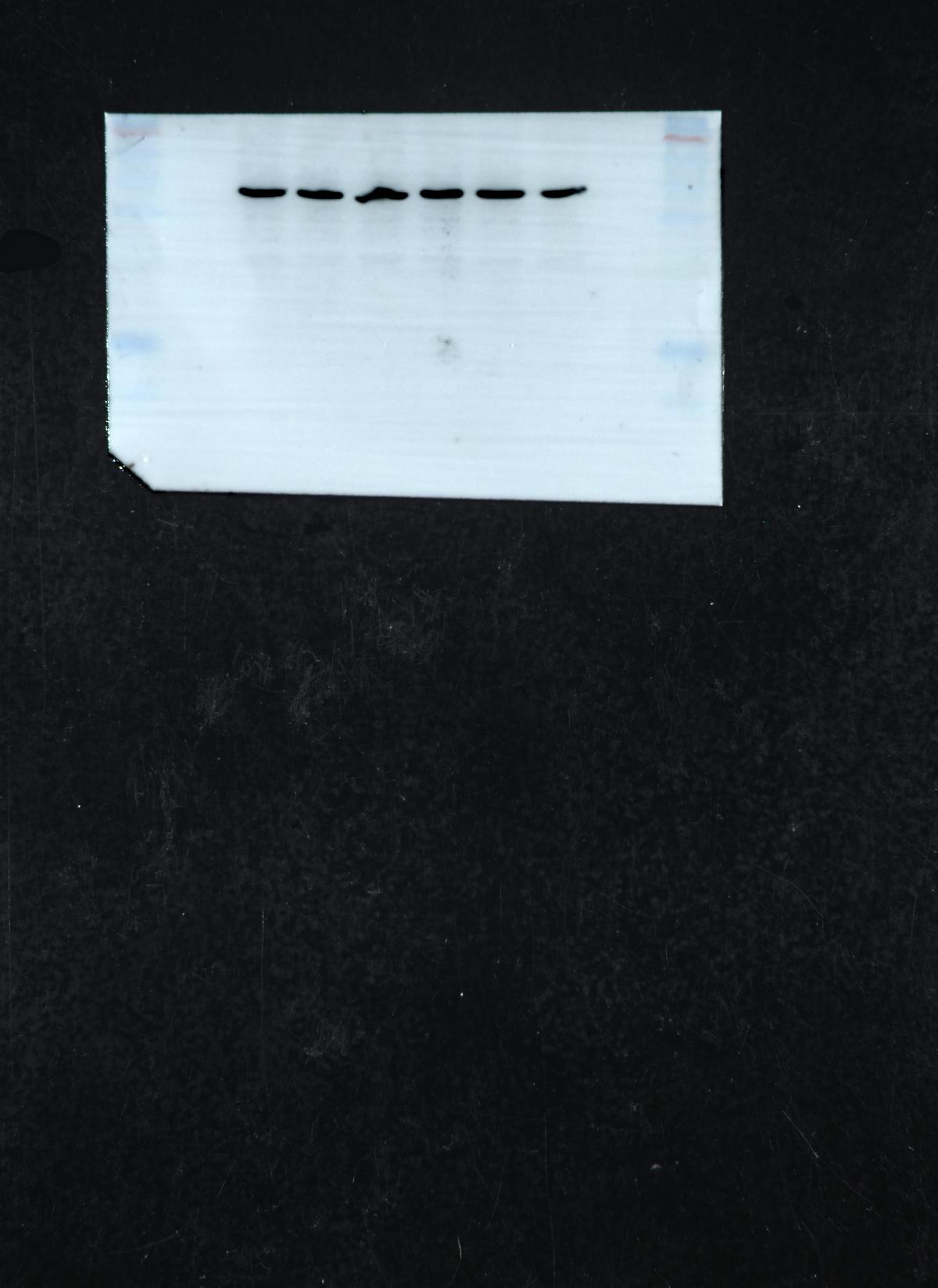

Supplement: Supplementary file 1 [file Data_Sheet_1.docx]
