# Supplementary material for: Effects of icariin as a feed additive on the reproductive function in bucks (Capra hircus)
Source: Front Vet Sci. 2024 Nov 6;11:1467947. doi: 10.3389/fvets.2024.1467947 (PMC11580527; doi:10.3389/fvets.2024.1467947)
Supplement: Supplementary file 2 [file Table_1.docx]

**Table S1. The qPCR primer sequences were used in the present study.**

| **Gene Name** | **Primers** | | **Accession number** |
| --- | --- | --- | --- |
|  | **Forward** **Reverse** | |  |
| *AR* | CAGTGGATGGGCTGAAAAAT | GGAGCTTGGTGAGCTGGTAG | XM_018043830.1 |
| *LHR* | GAAAGCACAGCAAGGAGACC | CAGTCACATTTCCCGTGATG | NM_001314279.1 |
| *FSHR* | GCAATTCTTTCATGGGGCTA | GAACGGATCCTGGTTCTTGA | NM_001285636.1 |
| *StAR* | TACTAAAGGAGCCGTGGATAAAG | CTACAAGTGGTAATGGTTGGGTT | XM_013975437.2 |
| *3β-HSD* | CTATGTTGGCAATGTGGC | ATCTCGCTGAGCTTTCTTAT | XM_013962472.2 |
| *TSPO* | ACCCAGTACATCCGTGGAGA | CCAGATGCGGTAGTTGAGCA | XM_018048872.1 |
| *INSL3* | GGCTGGAAGGACAACATCTC | GACAGAGGGTCAGCAGGTCT | NM_001285579.1 |
| *CYP17a1* | GCTCTTGGGTGTCTTTCTGC | GATGGGGCCATATTTTTCCT | NM_001314145.1 |
| *17β-HSD* | TCAGCTTCCAAGGCTTTTGT | CCACAGGTTTCGTCTCCAAT | XM_005684148.2 |
| *PGP9.5* | TGCTGAACAAAGTGCTGACC | GTTCCACAGGAGTTGCCAAT | XM_005681551.3 |
| *DDX4* | ATTTGCCTCTGGGAGGAGTT | AACCCTCTGTTCCGTGTTTG | XM_005694694.3 |
| *c-Kit* | GGATCACCGAGAAAGCAGAG | GCCACAAACGTCAAATCCTT | NM_001285724.1 |
| *SCP1* | CCCTGGAACTCAGGAAACAA | TGCTTCGAGCATTTTCTTCA | XM_018045949.1 |
| *SCP3* | GCTGGAAAGATTTGGAGCTG | ATCCCACTGCTGGAACAAAG | XM_018048175.1 |
| *AMH* | CGTGAGCTGAGCGTAGACCT | GAGGAGCTTGCCTGTGTAGG | XM_018050765.1 |
| *SOX9* | CTCAAGGGCTACGACTGGAC | CTCTCGTTCAGCAGTCTCCA | XM_018063905.1 |
| *β-actin* | CTGAGCGCAAGTACTCCGTGT | GCATTTGCGGTGGACGAT | NM_001314342.1 |
